# Book Reviews

**Published:** 1896-02

**Authors:** 


### Book Reviews, Etc.

A Manual of Syphilis and the Venereal Diseases. By James Nevins Hyde, A. M. M. D., Professor of Skin and Venereal Diseases, Rush Medical College, etc., and Frank H. Montgomery, M. D., Lecturer on Dermatology and Genito-Urinary Diseases, and Chief Assistant to the Clinic for Skin and Venereal Diseases, Rush Medical College, etc. Illustrated. Philadelphia: W. B. Saunders, 1895. Price, $2.50.

This book is to be commended as a practical guide to the subject. The descriptions are lucid and advice as to treatment sensible and practical. The illustrations, though not numerous, are apt. The sociological aspect of syphilis is most admirably presented and deserves to be widely read and seriously regarded. The language of the work, though sufficiently concise and expressive, is, it seems to us, a trifle too ornate for a text-book. Science does not concern itself with point lace and diamonds; the simplest garb best suits it.

An American Text-Book of Obstetrics. By numerous authors. Edited by Richard C. Norris, M. D. Illustrated. Philadelphia: W. B. Saunders, 1895. Price, $7.00.

This is the latest addition to the highly valuable series on practice, surgery, gynæcology and diseases issued by the house of Saunders. It is fully up to the high standard erected by the preceding volumes. All the more ambitious publications of this house are marked by an opulence of illustration and typographical excellence that is surprisingly out of proportion to the low price charged for them. The text is practically a series of monographs by fifteen of the best known gynæcologists and obstetricians in this country, as Henry J. Garriques, Richard C. Norris, Barton Hirst, Howard Kelly, R. L. Dickinson, of Brooklyn, and others equally well-known.

The possession of this book would go far toward simplifying the practice of obstetrics.

Principles and Practice of Medicine. By William Osler, M. D., F. R. C. P. (Lond.), Professor of Medicine, Johns Hopkins University, etc. *Second Edition.* New York: D. Appleton & Co., 1895. Octavo, pp. 1,143. Cloth, $5.50; sheep, $6.50; half morocco, $7.00, *Sold only by subscription.*

Professor Osler's book needs no introduction to the profession. There are certain points in the new edition that may be brought out to show the advantage of the revision. Sixty pages of new matter have been added. Among articles entirely new or recent are infantile scurvy, foot and mouth disease, bubonic plague, affections of the mesentery, eczema of the tongue, serum therapy, etc.

The advance of the second edition is sufficiently marked to warrant its addition even to the library of those who are in possession of the first.

Obstetrical Pocket-Phantom. By Dr. K. Shibata; preface by Prof. Franz von Winckel. With eight illustrations, one pelvis and two jointed manikins. Translated from the third edition by Ada Howard-Audenried, M. D. Philadelphia: P. Blakiston, Son & Co. Price, $1.00.

A little book designed by the author to act as an aid to the student of obstetrics, and to enable him to have ever at hand a miniature pelvis and manikin with which he can practically demonstrate the various positions of the child during the course of labor.

